# Methicillin resistance and selective genetic determinants of *Staphylococcus aureus* isolates with bovine mastitis milk origin

**Published:** 2017-06

**Authors:** Zohreh Ahangari, Masoud Ghorbanpoor, Masoud Reza Seifiabad Shapouri, Darioush Gharibi, Kiarash Ghazvini

**Affiliations:** 1 Department of Pathobiology, Faculty of Veterinary Medicine, Shahid Chamran University of Ahvaz, Ahvaz, Iran; 2 Department of Bacteriology and Virology, Faculty of Medicine, Mashhad University of Medical Sciences, Mashhad, Iran; 3 Antimicrobial Resistance Research Center, Bu-Ali Research Institute, Mashhad University of Medical Sciences, Mashhad, Iran

**Keywords:** *Staphylococcus aureus*, Virulence factor, Methicillin resistance, Bovine mastitis

## Abstract

**Background and Objectives:**

*Staphylococcus aureus* is one of the major causes of bovine mastitis, which can be transmitted from animals to humans. Methicillin-resistant *S. aureus* (MRSA) isolates are more attentive and if not treated promptly, they can cause death. The aim of this study was to determine the prevalence of methicillin resistance and frequency of selected virulence factors of *S. aureus* isolates with bovine mastitis milk origin in Ahvaz, southwest of Iran.

**Materials and Methods:**

During a two-year period (2014–2015), 75 *S. aureus* isolates were recovered from referred clinical and sub-clinical bovine mastitis milk samples. The isolates were phenotypically investigated for resistance to cefoxitin by Kirby-Bauer method. DNA were analyzed by PCR for *mecA* and selected genes that encode the virulence factors.

**Results:**

According to the results, the *spa*, *ebp*S, *fnb*, *bbp*, *clf*A, *clf*B, and *cna* genes were detected in 98.7, 97.3, 97.3, 86.7, 84, 84 and 65.3% of the isolates, respectively. Among the 75 isolates, only one (1.3%) isolate was methicillin-resistant. Totally, 39 isolates (50.7%) had all of these virulence factors except *mecA*. The results showed that 96% of the isolates had at least the *fnb*, *ebp*S and *spa* genes, signifying the noteworthy role of these genes in the pathogenesis of *S. aureus* bovine intra-mammary infection in this area.

**Conclusion:**

In the present study, the prevalence of *mecA* was relatively low, possibly indicating that cows do not play a significant role in community-acquired MRSA infection in this area. According to the results, studied virulence factors were somewhat prevalent, bearing in mind the probable risk of transmission of these isolates from cows to humans, especially those that are in close contact with infected cattle. The data presented here can be used for the introduction of a protective vaccine against this infection.

## INTRODUCTION

*Staphylococcus aureus* is a Gram-positive bacterium and one of the most important pathogenic coagulase positive staphylococci in humans and animals. This agent can cause various diseases in humans such as endocarditis, pneumonia, and life-threatening septicemia (1). In animals, *S. aureus* is responsible for a range of diseases, including mastitis, which cause considerable economic losses in the animal husbandry industry (2). *S. aureus* may be transmitted from animal or animal products to human (3).

*S. aureus* isolates which are resistant to semi-synthetic β-lactam antibiotics are known as MRSA. Usually, MRSA isolates possess a gene (*mecA*) to inactivate many antibiotics, including β-lactam drugs. Although the presence of *mecA* gene is closely related to the resistance to β-lactams, there are reports of *mecA*-negative MRSA (4). Some reports show (5, 6) that MRSA strains are expanding, and there are concerns about the treatment of the diseases caused by these isolates. Although the most common type of MRSA infection is hospital-acquired, but the community-acquired type of MRSA infection has been widely observed during the past decade (7).

It has been shown that *S. aureus* is a major cause of contagious bovine mastitis (8, 9). Antibiotic treatment of staphylococcal mastitis has often been ineffective, owing to the development of antibiotic-resistant strains, such as MRSA (10). Therefore, the introduction of new prevention methods is highly recommended.

*S. aureus* possesses many virulence factors. The majority of them are in the form of cell surface proteins. Matrix adhesion molecules, as one of the most common categories of surface proteins, mediating bacterial binding to host tissue and the extracellular matrix, such as collagen, fibronectin and fibrinogen (1). The binding is the first step of staphylococcal infection, so *S. aureus* surface components such as elastin binding protein (EbpS), collagen binding protein (Cna), fibronectin binding proteins A and B (FnbA, FnbB), fibrinogen binding protein (Fib), clumping factor A and B (ClfA, ClfB), and bone sialoprotein binding protein (Bbp) have an important role in colonization of this agent (1, 11, 12). The presence of these surface proteins is critical to the success of the organism as a commensal bacterium and as a pathogen (1). Staphylococcal protein A (SpA) is a multifunctional surface protein that is bound to an FC fragment of IgG of numerous mammalian species (human, mouse, rabbit, ruminants), causing immune evasion (13) and can act as a B-cell super-antigen (1, 14). It is, therefore, an important *S. aureus* virulence factor.

During the past decade, community-acquired MRSA infection has been increasingly observed, urging to highlight the role of animals in this type of infection. On the other hand, *S. aureus* can express many virulence factors, including surface proteins like adhesions that are covalently attached to peptidoglycan. Study of the prevalence of these proteins in any area is important in defining biomarkers that can be used for diagnostic purposes and in identifying potential vaccine specific for that region. In Iran, data on the genotypic and phenotypic characteristics of *S. aureus* isolates with bovine origin are very limited. The aim of this study was to determine the prevalence of some of the virulence factors of *S. aureus* isolates with bovine mastitis milk origin in Ahvaz, southwest of Iran. This data is essential for improving our understanding of pathogenesis and risk of interspecies transmission as well as adopting a proper preventive approach for this infection.

## MATERIALS AND METHODS

### Sample collection.

During a two-year period (2014–2015), referred clinical and sub-clinical bovine mastitis milk samples were inoculated on blood agar. After 24–48 h of incubation at 37°C, isolated colonies were examined by Gram staining under microscope for their morphological characters. The Gram-positive cocci were sub-cultured on blood agar plates. Suspected staphylococcal isolates were identifed by biochemical tests and oxidase negative, and catalase, coagulase and D-mannitol positive isolates were considered as probable *S. aureus* isolates (15).

### Molecular confirmation of suspected *S. aureus* isolates.

All probable *S. aureus* isolates and a standard MRSA strain (ATCC 33591) as a positive control were examined for the presence of *aro*A gene by PCR amplification with a pair of specific primers for *aro*A, which encodes 5-enolpyruvylshikimate-3-phosphate synthase (16). In order to extract the whole bacterial DNA, 3–5 colonies of each isolates were suspended in 200 microliter of sterile deionized water and heated at 100°C for 10 minutes. In order to remove the sediment and collect the supernatant, containing the crude extract of bacterial DNA for PCR amplification, the tubes were centrifuged at 10000 rpm for 5 minutes (17).

For *aro*A amplification, the reaction was performed in a final volume of 25 μl containing: 12.5 μl of the 2x Master Mix with 1.5 mM MgCl_2_ final concentration (Amplicon, Denmark), 1 μl (20 mM) of each *aro*A specific primers (Bioneer, South Korea), 3 μl DNA template (about 300 ng) and distilled water up to final volume of 25 μl in 0.2 ml reaction tube. The tubes were subjected to thermal cycling (Eppendorf, Mastercycler® 5330, Germany) using the program shown in Table 1. The PCR products were analyzed by gel electrophoresis using 1% agarose gel containing safe stain (Sinaclon, Iran) and visualized by trans-illuminator (Uvitec, England). The size of the amplified products was estimated by comparison with a DNA ladder (Fermentas, USA). The *aro*A positive isolates were considered as *S. aureus* and the DNA stored at −70°C, for analysis of selected virulence factors.

**Table 1.** List of primer sequences and thermal conditions used for amplification of selective genetic determinants in *S. aureus* isolates with bovine mastitis milk origin

**Gene**	**Primer**	**Sequence**	**Size**	**PCR Cycling Conditions**	**Reference**
*aro*A	Forward	AAGGGCGAAATAGAAGTGCCGGGC	1283	95°C-2 min; 40 × (95°C-60 s, 58°C-60 s, 72°C-90 s); 72°C-10 min.	16
	Reverse	CACAAGCAACTGCAAGCAT			

*fnb*	Forward	TACCATACTGCTGTGGATAGYGAA	822	95°C-5 min; 30 × (95°C-30 s, 60°C-30 s, 72°C-60 s); 72°C-5 min.	19
	Reverse	TTAGCTTACTTTTGGAAGTGTATCTTCTTC			
*bbp*	Forward	TCAAAAGAAAAGCCAATGGCAAACG	500		20
	Reverse	ACCGTTGGCGTGTAACCTGCTG			

*clf*A	Forward	ATTGGCGTGGCTTCAGTGCT	288	95°C-5 min; 30 × (95°C-30 s, 55°C-30 s, 72°C-30 s); 72°C-5 min.	21
	Reverse	CGTTTCTTCCGTAGTTGCATTTG			
*clf*B	Forward	ACATCAGTAATAGTAGGGGCAAC	204		
	Reverse	TTCGCACTGTTTGTGTTTGCAC			

*cna*	Forward	TTCACAAGCTTGGTATCAAGAGCATGG	450	95°C-5 min; 30 × (95°C-30 s, 60°C-30 s, 72°C-30 s); 72°C-5 min.	20
	Reverse	GAGTGCCTTCCCAAACCTTTTGAGC			
*spa*	Forward	ATATCTGGTGGCGTAACACCTGCTG	211		22
	Reverse	CGCATCAGCTTTTGGAGCTTGAGAG			

*ebp*S	Forward	GCAAGTAATAGTGCTTCTGCCGCTTCA	550	95°C-5 min; 30 × (95°C-30 s, 61°C-30 s, 72°C-45 s); 72°C-5 min.	20
	Reverse	CATTTTCCGGTGAACCTGAACCGTAGT			

*mecA*	Forward	GTAGAAATGACTGAACGTCCGATAA	307	95°C-5 min; 30 × (95°C-30 s, 50°C-30 s, 72°C-30 s); 72°C-5 min.	21
	Reverse	AATTCCACATTGTTTCGGTCTAA			

### Phenotypic detection of MRSA.

Screening of *S. aureus* isolates for detection of phenotypic methicillin-resistant strains was performed by antibiotic susceptibility test, using 30 μg cefoxitin discs (padtanteb, Iran) on Mueller Hinton Agar (Himedia, India) by disc diffusion (Kirby-Bauer) method, according to the guidelines of clinical and laboratory standards institute (CLSI) (18). According to CLSI recommendation, complete inhibition zone diameter of ≤21mm was considered as *S. aureus mecA* positive and those with an inhibition zone of >22 mm were as *mecA* negative. A standard MRSA strain (ATCC 33591) was used as positive control.

### Molecular detection of virulence genes.

The presence of seven virulence-related genes (*clf*A, *clf* B, *spa*, *fnb*, *bbp*, *ebp*S and *cna*) and a methicillin-resistant mediated (*mecA*) gene was investigated in 75 *S. aureus* isolates with bovine mastitis milk origin. To detect virulence factors, a duplex or monoplex PCR was performed with specific primers and thermal conditions listed in Table 1. The reaction mixture was the same, as for the amplification of *aro*A gene except for using specific primers of each selected gene(s). The PCR conditions for the amplification of genes are presented in Table 1. A standard *S. aureus* (ATCC 33591) and distilled water were used as positive and negative controls, respectively. The PCR products and a DNA ladder (Fermentas, USA) were loaded in a 1.5% agarose gel containing safe stain. The DNA bands were visualized using trans-illuminator (Uvitec, England).

## RESULTS

### Molecular confirmation of suspected *S. aureus* isolates.

**Fig. 1.**
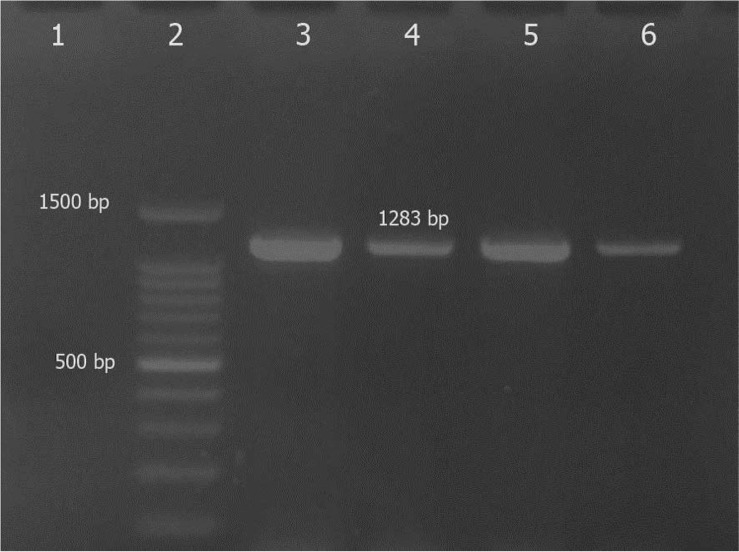
Agarose gel electrophoresis of the PCR products for amplification of *aro*A in *S. aureus* suspected isolates, Lanes 1 and 3: negative and positive controls, respectively, lanes 4–6, *aro*A positive isolates and lane 2, 100bp ladder

Out of 78 biochemically-suspected *S. aureus* isolates, 75 were *aro*A positive and confirmed as *S. aureus*. The products of PCR amplification of *aro*A gene in all isolates were uniform in size, approximately 1283bp (Fig. 1).

### Phenotype and genotype detection of methicillin-resistant *S. aureus*.

**Fig. 2.**
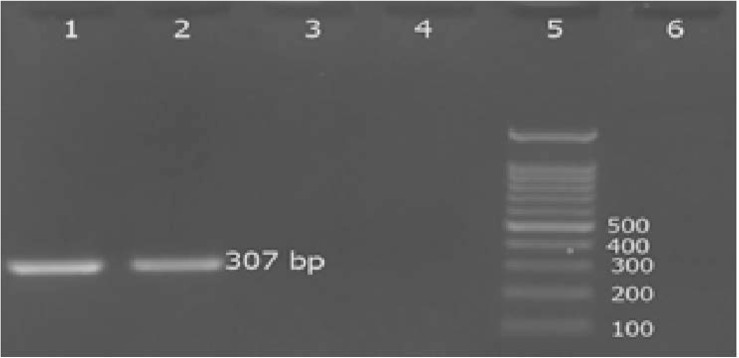
Agarose gel electrophoresis of the PCR products for amplification of *mecA* in suspected MRSA Lane 1: positive control *S. aureus* (ATCC 33591), lane 2: MRSA isolate, lanes 3, 4 negative *mecA* isolates, lane 6: negative control and lane 5, 100bp ladder.

Screening of 75 *S. aureus* isolates for detection of phenotypic methicillin-resistant strains showed that only 1 (1.3%) isolate was resistant to methicillin and had a complete inhibition zone diameter of 15 mm. PCR amplification of *mecA* confirmed the presence of *mecA* gene in this strain (Fig. 2).

### Frequency and distribution of virulence determinants.

**Fig. 3.**
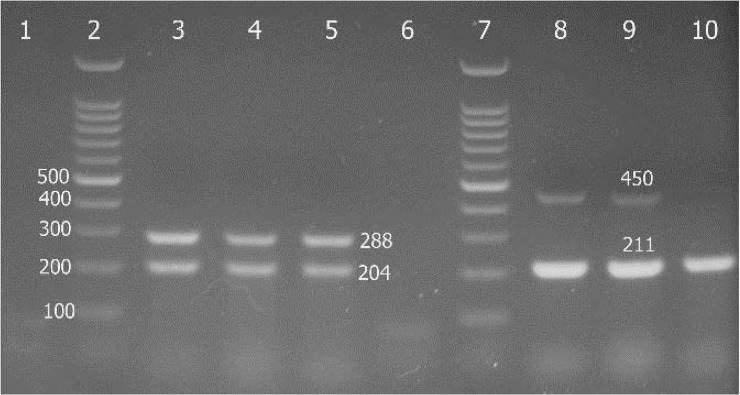
Agarose gel electrophoresis of the PCR products for detection of *clf*A, *clf*B, *cna* and *spa* gene of some *S. aureus* isolates, Lanes 1 and 3 negative and positive controls for *clf*A and *clf*B genes, lanes 4 and 5, *clf*A^+^ *clf*B^+^ isolates, lanes 6 and 8 negative and positive controls for *cna* and *spa* genes, lane 9 and 10, *cna*^+^ *spa*^+^ and *spa*^+^ isolates respectively, lanes 2 and 7, 100bp ladder.

**Fig. 4.**
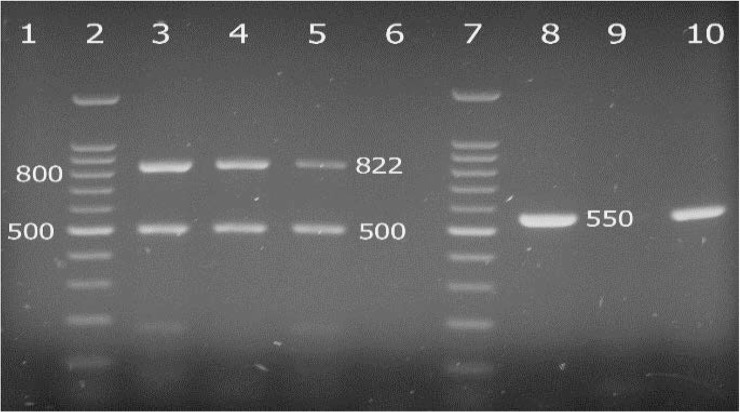
Agarose gel electrophoresis of the PCR products for detection of *fnb*, *bbp* and *ebp*S gene of some *S. aureus* isolates, Lanes 1 and 3 negative and positive controls for *fnb* and *bbp* genes, lanes 4 and 5, *fnb*^+^ *bbp*^+^ isolates, lanes 6 and 8 negative and positive controls for *ebp*S gene, lane 9 and 10, *ebp*S^+^ and *ebp*S^−^ isolates, respectively. lanes 2 and 7, 100bp ladder.

The results of the detection of virulence determinants (*clf*A, *clf* B, *spa*, *fnb*, *bbp*, *ebp*S and *cna*) in *S. aureus* isolates using PCR are shown in Figs. 3 and 4.

Evaluation of 75 *S. aureus* isolates for the presence of the *spa*, *clf*A, *clf* B, *bbp*, *fnb*, *can*, *ebp*S and *mecA* genes showed that the spa with 98.7% prevalence is the most common evaluated gene. The *fnb* and *ebp*S genes with an equal prevalence of 97.3% were also amongst the most prevalent genes of these isolates. The prevalence of *clf*A and *clf* B were equally 84% and *bbp* and *cna* with the respected prevalence of 86.7% and 65.3% were the least common analyzed genes. Accordingly, 38 isolates (50.7%) possessed all of these virulence factors except *mecA*. Based on the results, 96% of the tested isolates have at least the *fnb*, *ebp*S and *spa* genes, indicating the important role of these genes in the pathogenesis of bovine *S. aureus* mastitis in southwest of Iran. Among the 75 isolates, only 1 (1.3%) was both phenotypically and genotypically methicillin-resistant. The prevalence of selected virulence-related genotypes in tested isolates is listed in Table 2.

**Table 2.** Frequency of selected virulence-related genotypes in 75 isolates of *S. aureus* with bovine mastitis origin

**Genotypes**	**Number in 75 isolates (Percentage)**
*clf*A*^+^, clf*B*^+^, spa^+^, cna^+^, fnb^+^, bbp^+^, ebp*S*^+^*	38 (50.7)
*clf*A*^+^, clf*B*^+^, spa^+^, fnb^+^, bbp^+^, ebp*S*^+^*	17 (24)
*spa^+^, cna^+^, fnb^+^, bbp^+^, ebp*S*^+^*	6 (8)
*clf*A*^+^, clf*B*^+^, spa^+^, fnb^+^, ebp*S*^+^*	3 (4)
*spa^+^, cna^+^, fnb^+^, ebp*S*^+^*	2 (2.7)
*clf*A*^+^, clf*B*^+^, spa^+^, cna^+^, fnb^+^, ebp*S*^+^*	1 (1.3)
*clf*A*^+^, clf*B*^+^, fnb^+^, bbp^+^, ebp*S*^+^*	1 (1.3)
*clf*A*^+^, clf*B*^+^, spa^+^, fnb^+^, ebp*S*^+^*	1 (1.3)
*spa^+^, fnb^+^, bbp^+^, ebp*S*^+^*	1 (1.3)
*spa^+^, cna^+^, fnb^+^, ebp*S*^+^*	1 (1.3)
*clf*A^+^, *clf*B^+^, *spa*^+^, *ebp*S^+^	1 (1.3)
*clf*A^+^, *clf*B^+^, *spa*^+^	1 (1.3)
*spa*^+^, *fnb*^+^, *ebp*S^+^, *mecA*^+^	1 (1.3)
*spa*^+^, *fnb*^+^, *bbp*^+^	1 (1.3)

## DISCUSSION

*S. aureus* is the most frequent opportunistic pathogen in humans and one of the major causes of chronic and sub-clinical mastitis in dairy cattle. The present study was performed since there was limited data on virulence factors of *S. aureus* with bovine mastitic milk origin in southwest of Iran. The resistance of *S. aureus* against various antibiotics is a critical concern worldwide, but the methicillin resistance is the most well studied characteristic of this agent. MRSA usually spreads from human to human, but its transmission from animal to human is also possible (24, 25). Although MRSA has increasingly been recognized in farm animal populations in recent years, limited data is available with respect to the prevalence of MRSA in bovine mastitis milk sample in Iran. Our results suggest that there is a low prevalence (1.3%) of MRSA isolates in mastitis milk samples. This is consistent with studies of Virgin et al. (26) and Haran et al. (27) in USA and Hata et al. (28) in Japan, reporting the prevalence of 1–2% for MRSA. This sampling protocol, in addition to the selective double enrichment protocol for MRSA, may account for success in detecting MRSA and the higher rates of prevalence of MSSA isolates compared to previous studies. In contrast to our finding, Kumar et al. 2011 (29) in India reported a 9.3% (10 of 107 isolates) prevalence for *mecA* positive *S. aureus* isolates from bovine mastitis milk. Such relatively high prevalence (16.7%) has also been reported in studies of Havaei et al. (30) in Esfahan, Iran. The inconsistency may be due to the difference in methodology, time and location of the studied area. *S. aureus* can produce a group of other virulence factors, including adhesions factors needed for bacterial attachment to the host epithelial surfaces that is essential for the colonization, as the first step for initiation of infection. Therefore, identification of major virulence factors such as adhesins and determination of their frequency in isolates of *S. aureus* in any particular area is necessary for vaccine development. A vaccine that induce an efficient immune response to these proteins could be efficient against different *S. aureus* strains that use adhesins for colonization (31, 32). Recently, few studies have been performed regarding *S. aureus* derived from bovine or ovine mastitis. In this regard, Momtaz et al. (33) detected some virulence factors of *S. aureus* isolates from clinical and subclinical bovine mastitis samples in the center of Iran. Saei et al. (34) detected some virulence genes of *S. aureus* isolates with ovine mastitis origin in the northwest of Iran.

*S. aureus* protein A is a major virulence factor that is responsible for evasion from host immune responses (14). In the present research, the prevalence of this gene was 98.7%, showing the significant role of this gene in virulence of the tested *S. aureus* isolates. Consistent with this finding, in the studies of Momtaz et al. (33) in center of Iran, Klein et al. (35) in Brazil and Gogoi-Tiwari et al. (36) in Australia, the prevalence of this gene in *S. aureus* isolates in dairy bovine mastitis milk sample was reported to be 80.2%, 85.9% and 87.7%, respectively.

The *fnb* and *ebp*S with a similar prevalence of 97.3% were the second most prevalent virulence genes in the present study. In relative consistency with our finding, the frequency of *fnb* in comparable studies in other countries was reported to be 56% to 96% (35–37). Based on the study of Saei et al. (34) on *S. aureus* isolates from ovine mastitis in the northwest of Iran, the frequency of *fnb*A and *fnb*B were 90% and 77.7%, respectively. In contrast to the results of present study, the *ebp*S were detected in 25% of *S. aureus* isolates with bovine mastitis in Poland (38). However, consistent with our findings, Ikawaty et al. (37) found *ebp*S in all of the tested isolates. It seems that these two genes have a critical role in the pathogenesis of mastitis in animals.

*S. aureus* may interact with bone sialoprotein, a glycoprotein of bone, and dentine extracellular matrix to colonize at the site of infection (39). In our study, 86.9% of isolates possessed the bbp gene, but in similar research in Australia (34), this gene was detected in only 9.1% of isolates. However, Puacz et al. (40) reported the presence of 93.9% of this gene in *S. aureus* derived from bovine mastitis in central-eastern Poland.

Our result revealed that the frequency of *clf*A and *clf*B in examining isolates is equally 84%. The prevalence of *clf*A in isolates with bovine mastitis was reported to be 19%–100% and *clf*B was detected in 91.8%–92.9% of the isolates (33, 35–37).

Although *cna* is a major virulence factor in staphylococcal diseases, this gene had the lowest frequency (65.3%) in the tested isolates. Consistent with this finding, the prevalence of this gene in the study of Gogoi-Tiwari et al, (36), Ikawaty et al. (37) and Klein et al. (35) was reported to be 31.2%, 49% and 22.4%, respectively. Rhem et al. (41) and Elasri et al. (42) stated the role of the Cna protein in the osteomyelitis due to *S. aureus*, but according to the study of Elasri et al. (42), this protein does not have any important contribution in human skeletal muscle infections, though it contributes to human *S. aureus* keratitis. Consequently, *cna* may not play an important role in the pathogenesis of bovine mastitis due to *S. aureus*.

According to our findings, 75 isolates of *S. aureus* from bovine mastitis milk samples could be grouped in 14 genotype profiles, based on the selected following genes (Table 2) and the most frequent profile with a prevalence of 50.7% belongs to the one that possessed at least 7 of 8 selected genes. These findings confirm the inconsistency of this bacterium in the southwest of Iran and help us in establishing a suitable protective approach to decrease the occurrence of this important disease in dairy farms. Similarly, based on 7 genes, Coelho et al. (43) reported 27 different profiles for *S. aureus* isolates recovered from intra-mammary infections amongst 25 dairy cattle farms in Rio de Janeiro, Brazil.

In conclusion, although *S. aureus* isolates with bovine mastitis origin differ in their genetic profile in the southwest of Iran, 96% of all isolates simultaneously possessed *fnb*, *ebp*S and *spa* genes, representing the significant role of these genes in pathogenesis of bovine intra-mammary infection due to prevalence of *S. aureus* in this area. This can help in the exploration and control of *S. aureus* mastitis in bovine dairy farms. Whether the comparative prevalence of selected virulence genes of *S. aureus* is applicable to the introduction of a protective vaccine against staphylococcal bovine mastitis, cannot be deducted from the data existing in this research. However, it may be useful bearing in mind this evidence, in the development of an effective vaccine against this important disease. On the other hand, it seems that in tested area, the risk for the spread of MRSA from bovine sources into the human population will be low. However, high risk persons who are in close contact with MRSA-infected cattle, including veterinarians, slaughterhouse staff, and livestock workers, may get infected with bovine mastitis milk.
